# University of Maryland

**Published:** 1860-05

**Authors:** 


*University of Maryland.*—Dr. Edward Warren, of Edenton, North Carolina, has been appointed to fill the Chair of Materia Medica and Therapeutics vacated by the death of Prof. Frick, and Dr. Farnandis has been named as Demonstrator of Anatomy in stead of the late Dr. Berwick B. Smith. Dr. Warren contributed a number of articles to the MONTHLY some years since, and is now the editor of a medical journal published in North Carolina. In assuming the duties of his Chair, he bears with him our best wishes for success.

